# HAb18G/CD147 Regulates Vinculin-Mediated Focal Adhesion and Cytoskeleton Organization in Cultured Human Hepatocellular Carcinoma Cells

**DOI:** 10.1371/journal.pone.0102496

**Published:** 2014-07-17

**Authors:** Qiang Liang, Qing Han, Wan Huang, Gang Nan, Bao-Qing Xu, Jian-Li Jiang, Zhi-Nan Chen

**Affiliations:** 1 Cell Engineering Research Centre and Department of Cell Biology, State Key Discipline of Cell Biology, Fourth Military Medical University, Xi’an, Shaanxi, China; 2 Department of Clinical Immunology, Xijing Hospital, Fourth Military Medical University, Xi’ an, Shaanxi, China; The University of Hong Kong, Hong Kong

## Abstract

Focal adhesions (FAs), integrin-mediated macromolecular complexes located at the cell membrane extracellular interface, have been shown to regulate cell adhesion and migration. Our previous studies have indicated that HAb18G/CD147 (CD147) is involved in cytoskeleton reorganization and FA formation in human hepatocellular carcinoma (HCC) cells. However, the precise mechanisms underlying these processes remain unclear. In the current study, we determined that CD147 was involved in vinculin-mediated FA focal adhesion formation in HCC cells. We also found that deletion of CD147 led to reduced vinculin-mediated FA areas (*P*<0.0001), length/width ratios (*P*<0.0001), and mean intensities (*P*<0.0001). CD147 promoted lamellipodia formation by localizing Arp2/3 to the leading edge of the cell. Deletion of CD147 significantly reduced the fluorescence (*t*_1/2_) recovery times (22.7±3.3 s) of vinculin-mediated focal adhesions (*P*<0.0001). In cell-spreading assays, CD147 was found to be essential for dynamic focal adhesion enlargement and disassembly. Furthermore, the current data showed that CD147 reduced tyrosine phosphorylation in vinculin-mediated focal adhesions, and enhanced the accumulation of the acidic phospholipid phosphatidylinositol-4, 5-bisphosphate (PIP2). Together, these results revealed that CD147 is involved in vinculin-mediated focal adhesion formation, which subsequently promotes cytoskeleton reorganization to facilitate invasion and migration of human HCC cells.

## Introduction

Migration is a critical step in tumor invasion and metastasis and involves decreased cell adhesion, cytoskeleton remodeling, extracellular matrix degradation and protrusion formation. Focal adhesions (FAs) are macromolecular complexes formed by various junctional proteins. They are located at connecting sites for integrin-mediated cell matrix adhesion, and participate in cell adhesion, migration and survival [1], [2].

FAs regulate the spatial and temporal dynamic organizational states of F-actin polymerization, which creates tension to pull the cell body forward [3], [4]. With the dynamic processes of assembly/disassembly, FAs alter cell size and position to control cell migration [5].

CD147 has been reported to be a cancer marker which belongs to the immunoglobulin superfamily and overexpressed in HCC cells [6]. CD147 plays important roles in cellular processes of adhesion, invasion, migration, and extracellular matrix degradation [7]–[9]. Our previous studies indicated that CD147 up-regulates the activities of integrins α3β1 and α6β1, leading to cytoskeleton rearrangement and changes in cell morphology through the FAK-paxillin and FAK-PI3K-Ca^2+^ signaling pathways, and subsequently enhances invasion and metastasis [10], [11]. We also showed that CD147 positively correlates with Rac1 activity, which contributes to the formation of lamellipodia and mesenchymal movement of HCC cells [12]. Deletion of CD147 reduced the number of focal adhesions and rearrangement of the cytoskeleton in HCC cells [10], [13]. However, the precise role of CD147 in the regulation of FA formation and subsequent cytoskeleton reorganization to promote invasion and metastasis is not well understood.

Vinculin links adhesion plaques to F-actin fibers by initiating the formation of bundled actin fibers or by remodeling existing microfilaments [14]. Vinculin knockout enhances the migration of mouse embryonic fibroblasts, impairs the formation of FAs, and decreases the strength of adhesion to ECM [15].

The aim of this study was to reveal the precise role of CD147 in vinculin-mediated FA morphology, cytoskeleton reorganization, and lamellipodia formation.

## Materials and Methods

### Cell culture [10]

Human SMMC-7721 HCC cells were obtained from the Institute of Cell Biology, Academic Sinica, Shanghai, China. K7721 cells (CD147 is stably knocked out in SMMC-7721 cells) was developed in our laboratory. All cells were maintained in RPMI 1640 medium (Gibco, New York, USA) supplemented with 10% FBS, 1% penicillin/streptomycin and 2% L-glutamine at 37°C in a humidified atmosphere with 5% CO_2_. The following antibodies were used: phospho-tyrosine mouse mAb (Cell Signaling, Boston, MA, US), anti-APR3 mAb (Sigma, St. Louis, MO, US), PIP2 (Abcam, Cambridge, MA, US). All cell imaging and immunoblotting were performed with cells cultured on a thin layer of Matrigel. Two µl of mouse Matrigel (BD Bioscience, Franklin Lakes, NJ, USA) was diluted with RPMI 1640 medium for a total volume of 200 µl, and added into the bottom of a 35 mm diameter dish (NEST, Wuxi, Jiangsu, China) for each culture. Cells were seeded on top of the Matrigel in RPMI 1640 containing 10% serum and cultured for 16 h.

### Co-immunoprecipitation [10]

CD147 interaction with vinculin in native cells was detected with a ProFound™ Mammalian Co-Immunoprecipitation Kit (Pierce, Rockford, IL, US), according to the manufacturer’s instructions. Briefly, SMMC-7721 cells (1×10^6^) were lysed with M-per reagent. The lysate was collected and placed on coupling columns that were pre-bound with 50 µg of the mouse anti-human CD147 monoclonal antibody (1 mg/ml) (mAb) HAb18 (developed in our lab) or a mouse anti-human vinculin monoclonal antibody (0.2 ml) (Sigma v9131, St. Louis, Missouri, US), and mouse IgG antibody (1 mg/ml) as used as a control. Columns were washed with co-immunoprecipitation buffer. Bound proteins were eluted from the coupling gel with elution buffer, and aliquots of the eluent were analyzed by Western blotting using the vinculin mAb and HAb18 mAb.

### Immunofluorescence [13]

Cells were allowed to attach for 3 h to dishes pre-coated with Matrigel. Cells were then fixed with 4% formaldehyde in PBS, permeabilized with 0.1% Triton X-100 and blocked with 1% BSA (Beyotime, Shanghai, China) in PBS for 30 min. Dishes were incubated with the vinculin antibody (Sigma, St. Louis, MO, US) at a 1∶500 dilution for 2 h and with rhodamine-phalloidin (Molecular Probes, New York, US) at a 1∶100 dilution in PBS for 1 h. Antibody-treated cells were washed in PBS and incubated with an Alexa 594 goat anti-mouse secondary antibody (Pierce, Rockford, IL, US) at a 1∶400 dilution in PBS for 1 h. Cell nuclei were stained with DAPI (Vector Labs, CA, US) for 5 min. Cells probed with rhodamine-phalloidin were washed. Finally, cells were observed with an A1 confocal microscope (Nikon, Tokyo, Japan).

### Images Analysis [5]

The image analysis was performed using Nikon NIS-Elements software (Nikon, Tokyo, Japan). An automeasurement method in NIS-Elements software was provided for efficient parameter extraction and statistical analysis. The method is based on fluorescence intensity contrast, pixel by pixel, in a single channel. First, the confocal merged picture was split into a different channel. Then, the individual FAs were detected by an automeasurement method in single channel image. The area and intensity option buttons of software settings panel were adjusted to select the region of interested. The software settings and all selected FAs were recorded. Finally, all qualified FAs were automatic numbered, and examined by NIS-Elements software. The values and all information were exported as excel files. Statistical significance was determined using Student’s *t*-test. GraphPad Prism software (Cricket Software, Philadelphia, PA) was used for the above analyses, and P values less than 0.05 were considered significant.

### RNA interference

The double-stranded siRNA was purchased from Shanghai GenePharma (Shanghai, China). The sequence for si-HAb18G were as described previously [7]. HCC cells were transfected with siRNA using LipofectAMINE 2000 reagent (Invitrogen, Carlsbad, CA, US) according to the manufacturer’s instructions. The silencer negative control siRNA (snc-RNA) was used as a negative control under similar conditions.

### Western Blotting [10]

HCC cells were harvested in lysis buffer and a BCA Protein Assay Kit (Pierce, Rockford, IL, US) was employed to determine the total protein concentration. Equal amounts of protein were separated by SDS-PAGE on a 12% polyacrylamide gel and then transferred to a polyvinylidene fluoride (PVDF) microporous membrane (Millipore, Boston, MA, USA). After blocking with 5% non-fat milk, the membrane was incubated for 1 h at room temperature with the indicated antibody. Tubulin was chosen as an internal control, and the blots were probed with mouse anti-tubulin mAb (Santa Cruz Biotechnology, Santa Cruz, CA, USA).

### Cell adhesion assay

Ninety-six-well culture plates were coated with Matrigel (BD Bioscience, Franklin Lakes, NJ, USA), blocked with PBS containing 2% BSA diluted in PBS at 4°C overnight, washed with PBS, blocked with 100 µl of 2% heat-denatured BSA at RT for 2 h, and rinsed three times with PBS. Cells in serum-free RPMI1640 medium were added into the 96-well culture plates (5×10^5^ cells/well) and permitted to attach for 30 min. After removing non-adherent cells, attached cells were fixed in 10% paraformaldehyde at RT for 30 min and then stained with 1% toluidine blue for 60 min. Plates were gently washed with tap water and air-dried. One hundred µl of 2% SDS solution were applied for 20 min, and the absorbance was measured at 620 nm using a microtiter plate reader [16] (BioTek Synergy2, Shanghai, China).

### Plasmid construction and transfection

Full-length vinculin (residues 1–1066) lacking a stop codon was PCR-amplified from SMMC-7721 cells, and cloned into DsRed1-N1 (Clontech, Mountain View, CA, US) or pEGFP-N1 (Clontech, Mountain View, CA, US). pEGFP-CD147 was developed in our lab and was used as previously described [17]. DsRed1-N1 and pEGFP together were used as a FRET pair.

The DsRed1-N1-vinculin plasmid containing full-length vinculin (residues 1–1066) was used as a template for vinculin mutant construction. Tyrosines 100 and 1065 were replaced with phenylalanines to create three mutations (Y100F, Y1065F, Y100/1065F) using the QuikChange Multi Site-Directed Mutagenesis Kit (Stratagene, Santa Clara, CA, US) according to the manufacturer’s instructions. All mutations were confirmed by sequencing (BGI Tech, Beijing, China).

Site-directed mutation of N-glycosylation sites was performed with the QuikChange Lightning Multi Site-Directed Mutagenesis Kit (Stratagene, Santa Clara, CA, US) to introduce asparagine-to-glutamine mutations in the expression plasmid pcDNA3-CD147 encoding the CD147 protein. We used the following primers: N44Q (sense-ctcacctgctccttgcaggacagcgccacagag; antisense-ctctgtggcgctgtcctgcaaggagcaggtgag), N152Q (sense-acaaggccctcatgcagggctccgagagcag; antisense-ctgctctcggagccctgca tgagggccttgt) and N186Q (sense-ccagtaccggtgccagggcaccagctcca; antisense-tggagctgg tgccctggcaccggtactgg). Triple mutants were produced according to the manufacturer’s instructions. Introduction of these mutations into cDNAs was verified by DNA sequencing (BGI Tech, Beijing, China). Cells were seeded on Matrigel-coated 35 mm dishes. Transfection was performed using Lipofectamine 2000 reagent (Invitrogen, Carlsbad, CA, US) according to the manufacturer’s instructions.

### FRET measurement

SMMC-7721 cells were plated into 35 mm culture dishes. Fluorescent images for FRET samples, donor samples, and acceptor samples were captured 16 h after transfection using an A1 confocal microscopy (Nikon, Tokyo, Japan) with 3-FRET filter cubes for EGFP/DsRed: EGFP (488/515 nm), DsRed (543/585 nm), and FRET (488/585 nm) [18]. Regions of interest were selected for all non-saturated fluorescent cells in any given field. The background was subtracted from all images before analysis. Donor (pEGFP-CD147) and accepter (DsRed1-N1-vinculin) images were utilized to obtain CoA and CoB data for the next FRET calculation with NIS-Elements software (Nikon, Tokyo, Japan). FRET calibration and net FRET were calculated with analysis software (Nikon, Tokyo, Japan). FRET analysis was performed using a three-filter setup system based on the sensitized emission method. Each experiment was repeated at least three times, and similar results were obtained for each [19].

### FRAP

A confocal microscope (Nikon A1, Tokyo, Japan) with a 60×1.40 NA Plan Apochromat oil objective (Nikon, Tokyo, Japan) equipped with a 488 nm laser line (Nikon, Tokyo, Japan) under the control of NIS-Elements software was used for the FRAP experiments. Cells were plated onto Matrigel-coated glass bottom 35 mm dishes. Cells expressing the indicated vinculin EYFP-tagged constructs were then imaged at 37°C in RPMI1640 medium. Initial fluorescence intensity was measured at low laser power followed by photobleaching of FAs at 100% laser power for 10 s. Fluorescence recovery was then followed with low laser power at 3 s intervals until fluorescence intensities recovered to a plateau. Corrected recovery fluorescence intensities were normalized to the pre-bleach intensity. The intensity of the recovery curve was calculated according to Phair’s single normalization method [20]. To determine the τ_1/2_ of recovery, normalized recovery data were fitted to a single exponential equation [21], and the τ_1/2_ of recovery was calculated from the recovery curve.

### Cell spreading assay

For spreading assays [22], cells (1×10^5^) were seeded onto Matrigel-coated 35 mm dishes in RPMI 1640 with 10% FBS, and photographs of at least 6 random microscope fields (Nikon A1 confocal microscope, Tokyo, Japan) were taken 60 min (for spreading) and 24 h (for morphology) after seeding. Cell area was measured for at least 6 cells, and the spread area was calculated using Nikon software.

## Results

### CD147 activates the Arp2/3 complex for lamellipodia assembly

**Figure 1:**
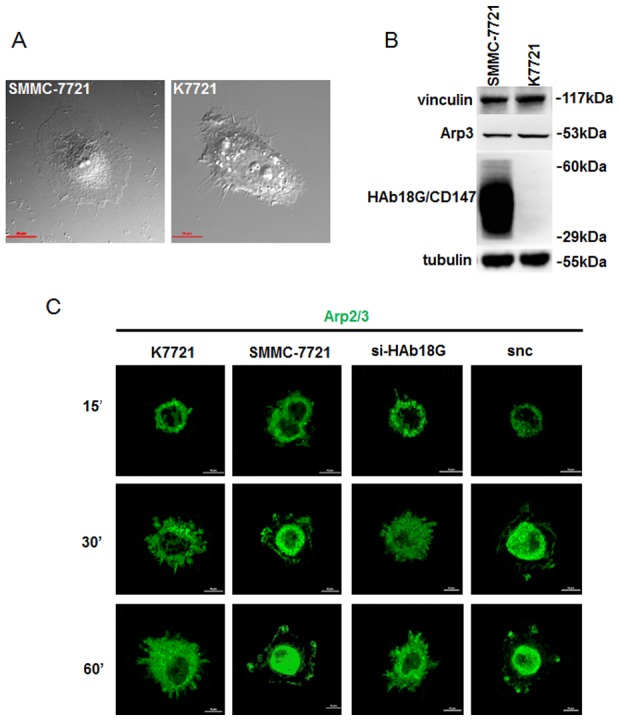
Effects of alteration of CD147 expression on vinculin levels and protrusion formation in HCC cells. (A) Differential interference contrast (DIC) microscopy of protrusion formation in SMMC-7721 and K7721 cells. Scale bar = 20 µm. (B) Western blots of CD147, Arp3, and vinculin in SMMC-7721 and K7721 cells. (C) Time-lapse immunofluorescence imaging of Arp2/3 distribution during protrusion formation. SMMC-7721 and K7721 cells were harvested at 70–80% confluency and seeded onto thin Matrigel-coated 35 mm dishes. The Arp2/3 complex was detected by immunofluorescence after 15 min, 30 min, and 60 min of culture. Scale bar = 10 µm.

To examine the role of CD147 in cancer cell motility, we used SMMC-7721 cells in which CD147 was highly expressed, and K7721 cells in which CD147 expression was knocked out (Fig. 1B). Using a differential interference contrast (DIC) microscope, we observed that knockout of CD147 expression in K7721 cells significantly attenuated lamellipodia formation, and resulted in the formation of filopodial protrusions at the leading edge compared to SMMC-7721 cells (Fig. 1A). Western blots showed that vinculin and Arp2/3 expression were not affected by the presence or absence of CD147 (Fig. 1B). To further elucidate the role of Arp2/3 in protrusion formation, we used a time-lapse immunofluorescence imaging assay to localize the Arp2/3 complex. As shown in Fig. 1C, the Arp2/3 complex was activated and accumulated in lamellipodia at the leading edge of SMMC-7721 cells. However, deletion of CD147 resulted in a diffuse distribution of the Arp2/3 complex in the cytoplasm of K7721 cells, and filopodia appeared at the cell edge. These results indicate that CD147 promotes Arp2/3 activation for lamellipodia assembly.

### CD147 promotes focal adhesion enlargement and affects cytoskeleton organization

**Figure 2:**
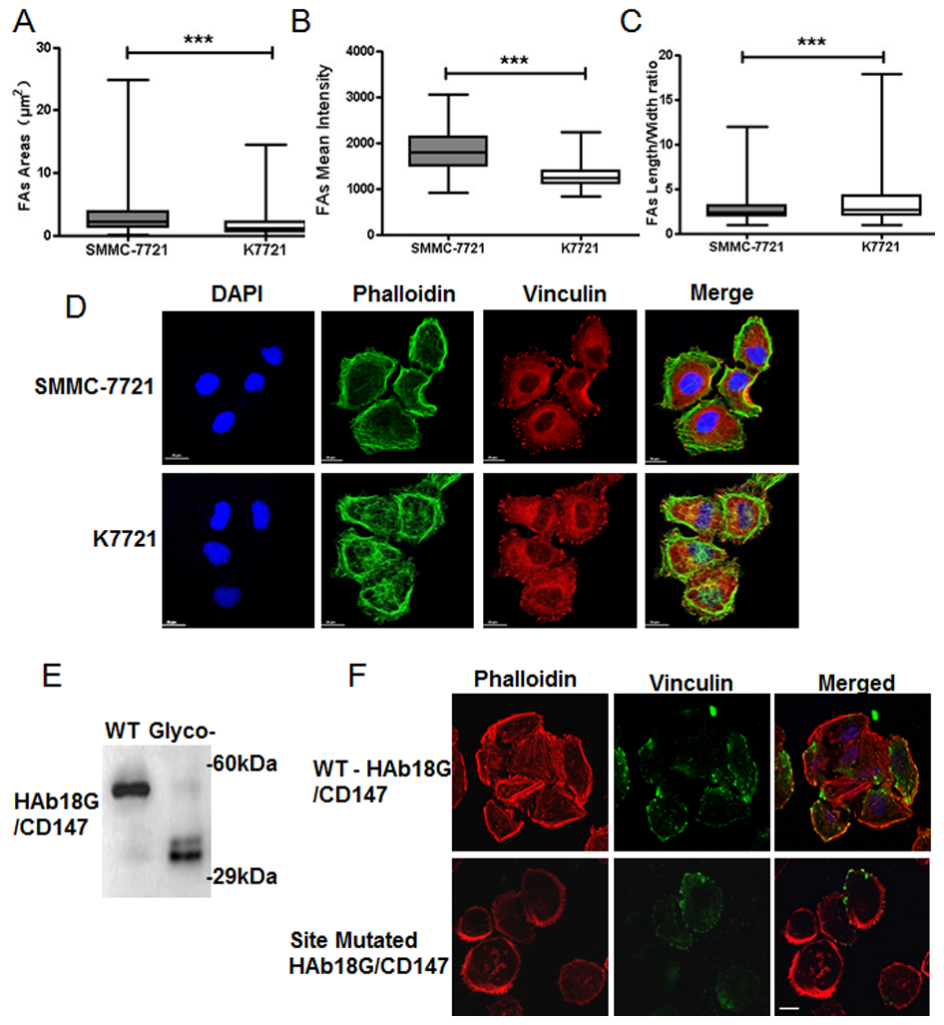
Effects of CD147 on FA morphology and cytoskeleton organization in HCC cells. (A) Endogenous vinculin was immunofluorescently stained. Vinculin-mediated FA areas were quantitatively analyzed with Nikon NIS-Elements software. Error bars represent standard errors of the means. ***P<0.0001 (Student’s *t*-test). (B) FA length/width ratios were analyzed with Nikon NIS-Elements software. Error bars represent standard errors of the means. ***P<0.0001 (Student’s *t*-test). (C) FA mean intensity was analyzed with Nikon NIS-Elements software. Error bars represent standard errors of the means. ***P<0.0001 (Student’s *t*-test). (D) Confocal microscopy images of FA and the cytoskeleton in SMMC-7721 and K7721 cells. Scale bar = 20 µm. (E) Cell lysates from K7721 cells expressing WT-CD147 and glycosylation site-mutated CD147 were analyzed by Western blotting with an anti-CD147 antibody. (F) Confocal microscopy analysis demonstrated the cytoskeleton rearrangement and focal adhesion redistribution induced by N-glycan deletion. Cells transfected with WT-CD147 and site-mutated CD147 were stained with rhodamine-phalloidin (red) and anti-vinculin antibody (green). Scale bar = 20 µm.

As shown in Fig. 2A, FA areas clearly decreased when CD147 was knocked out in K7721 cells (*P*<0.0001). However, FA length/width ratios significantly increased in K7721 cells compared to SMMC-7721 cells (*P*<0.0001) (Fig. 2B). Deletion of CD147 also reduced FA mean intensity in K7721 cells (*P*<0.0001) (Fig. 2C). These results indicate that CD147 promotes FA enlargement in HCC cells.

To assess the effects exerted by CD147 on cytoskeleton organization, F-actin stress fibers were labeled with rhodamine-phalloidin. As shown in Fig. 2D, larger bundles of actin were well organized, and lacked small actin branches in SMMC-7721 cells. However, actin architecture was disorganized in K7721 cells with small bundles and many tiny actin branches distributed in the central area of the cells. These observations demonstrate that CD147 affects the organization of the actin filament network.

CD147 is a highly N-glycosylated transmembrane protein with an ectodomain consisting of two regions exhibiting characteristics of the immunoglobulin (Ig) superfamily [23]. To elucidate the role of CD147 N-glycosylation in cytoskeleton rearrangement and focal adhesion distribution, we produced N-glycosylation defective mutants by substituting Asn with Gln via site-directed mutagenesis for all three predicted N-glycosylation sites. Mutation efficacy was determined by immunoblotting. WT-CD147 and its glycosylation mutant were separately transfected into K7721 cells. Mutation of all three N-glycosylation sites caused a decrease in molecular weight (∼30 kDa) compared to WT-CD147 (Fig. 2E). FAs marked by vinculin were located at the edges of cells expressing WT-CD147 or in lamellipodia protrusions (Fig. 2F), whereas the CD147 glycosylation mutant resulted in the reduction and disorganization of F-actin stress fibers.

### CD147 inhibits tyrosine phosphorylation of vinculin and stimulates the accumulation of PIP2 to promote FA formation

Vinculin is an important FA protein that is tyrosine phosphorylated (pTyr). This post-translational modification is important for maintaining its conformation, activity, and localization [24].

**Figure 3:**
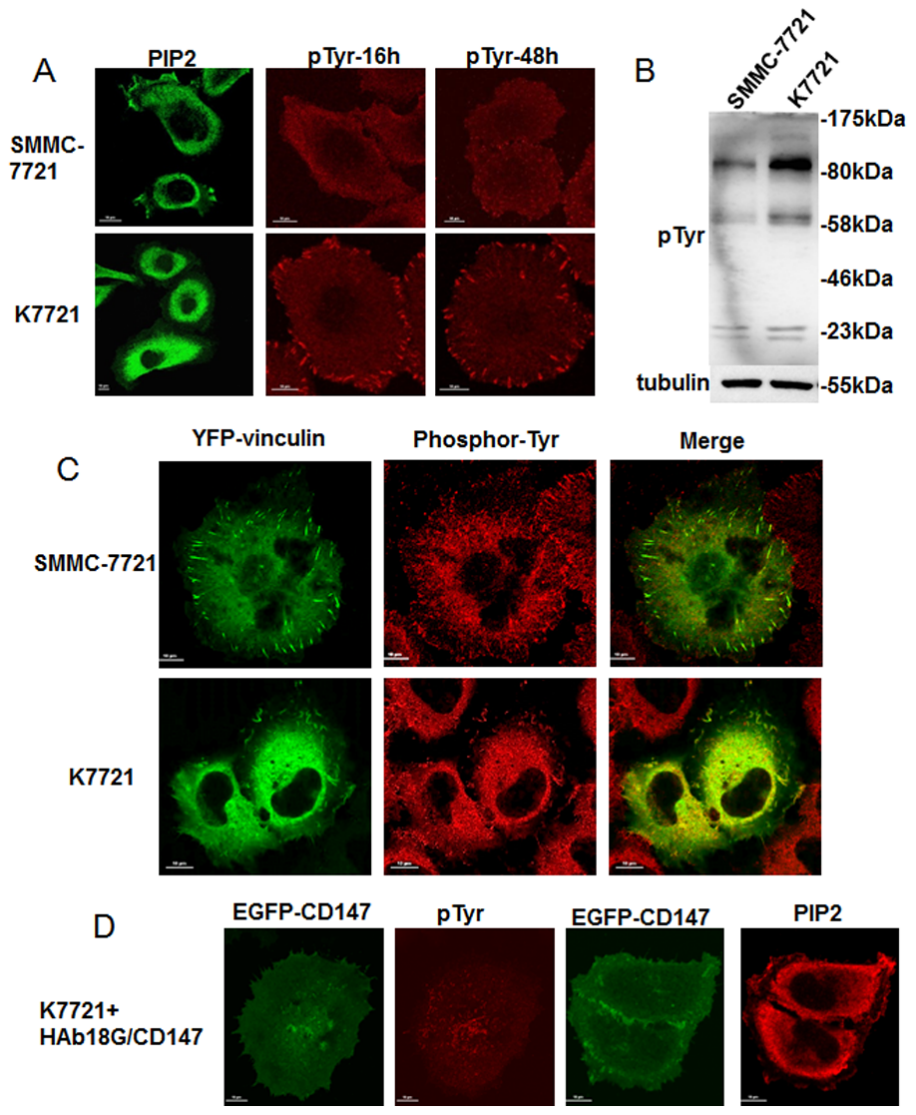
CD147 effects on FA tyrosine phosphorylation, and expression and localization of PIP2. (A) Confocal microscopy images of pTyr to visualize FAs (red) and PIP2 (green). Immunofluorescence images of pTyr were detected at two time points (16 h and 48 h). Scale bars = 10 µm. (B) Western blots of tyrosine phosphorylation in SMMC-7721 and K7721 cells. (C) Confocal microscopy images of tyrosine-phosphorylated vinculin in SMMC-7721 and K7721 cells. YFP-vinculin was transfected into HCC cells. After culture for 16 h, tyrosine phosphorylation levels were examined via an immunofluorescence assay. Scale bars = 10 µm. (D) The pEGFP-CD147 plasmid was transfected into K7721 cells. PIP2 (red) and pTyr (red) were detected via an immunofluorescence assay. Scale bars = 10 µm.

Utilizing an immunofluorescence assay, we detected high levels of tyrosine phosphorylation in K7721 cells after culture for 16 h and 48 h (Fig. 3A). No phosphorylated tyrosine was detected in SMMC-7721 cells. As shown in Fig. 3B, deletion of CD147 led to an approximate 3-fold increase in the band between 80–175 kDa. The band at about 58 kDa also increased about 2-fold. Western blot results indicated that total tyrosine phosphorylation levels were higher in K7721 cells than that in SMMC-7721 cells. To study the tyrosine-phosphorylated vinculin levels, we transfected YFP-vinculin into both SMMC-7721 and K7721 cells. As shown in Fig. 3C, the phosphorylation levels of vinculin were higher in K7721 cells than in SMMC-7721 cells.

PIP2 is essential for vinculin activation and acts by interrupting head-tail vinculin interactions [25]. We used an immunofluorescence assay to determine the location of PIP2 in HCC cells. As shown in Fig. 3A, PIP2 accumulated and localized to the periplasmic space of SMMC-7721 cells. However, PIP2 was redistributed and localized to the central cytoplasmic space in K7721 cells. To confirm these results, the pEGFP-CD147 plasmid was transfected into K7721 cells. As shown in Fig. 3D, PIP2 relocated in the periplasmic space of K7721 cells after CD147 re-expression, and tyrosine-phosphorylated vinculin-mediated FAs were also significantly reduced.

Taken together, these results indicate that CD147 inhibits tyrosine phosphorylation of vinculin and stimulates the accumulation of PIP2 to promote FA formation.

### CD147 interacts with vinculin in HCC cells

**Figure 4:**
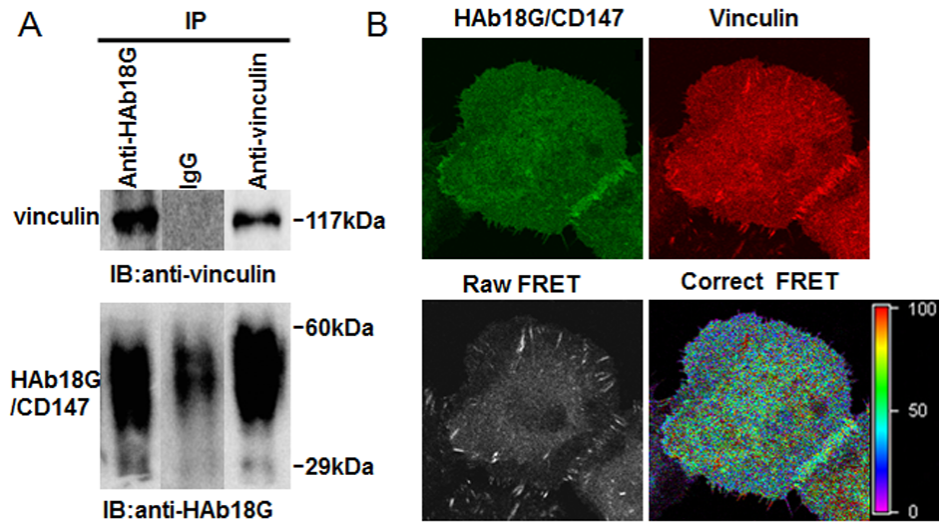
CD147 interaction with vinculin in HCC cells. (A) CD147 immunoprecipitated with vinculin. Lysates of SMMC-7721 cells were subjected to immunoprecipitation with an anti-CD147 or anti-vinculin antibody pre-bound coupling gel. Vinculin and CD147 were detected in immune complexes by immunoblotting (IB) analysis. Mouse IgG was used as a negative control. (B) The interaction between vinculin and CD147 was analyzed with FRET imaging. Two individual dishes of SMMC-7721 cells were transfected with DsRed1-N1-vinculin or pEGFP-CD147 as the acceptor and donor samples, respectively. Both DsRed1-N1-vinculin and pEGFP-CD147 were transfected into SMMC-7721 cells together as the FRET sample. Using the donor and acceptor samples, CoA and CoB were automatically calculated with FRET software. The correct FRET is representative of the interaction affinity. The FRET ratio is shown on the right.

To investigate the interaction between CD147 and vinculin, we undertook co-immunoprecipitation assays. As shown in Fig. 4A, CD147 co-immunoprecipitated with vinculin in SMMC-7721 cells. We then constructed two plasmids, DsRed1-N1-vinculin and pEGFP-CD147, and utilized the FRET technique to test this interaction in SMMC-7721 cells. As shown Fig. 4B, the highest FRET ratio was observed largely in proximity to vinculin-mediated FAs, after subtracting the background. Vinculin has two important phosphorylation sites (pTyr100 and pTyr1065). We constructed vinculin mutants for tyrosines 100, 1065, and 100/1065 and named these Y100F, Y1065F and Y100/1065F, respectively. When these vinculin mutants were paired with pEGFP-CD147 as a FRET probe, the FRET ratio was significantly reduced (Fig. S1). These results indicate that Tyr100 and Tyr1065 are important for the interaction between vinculin and CD147.

Taken together, our results suggest that CD147 interacts with vinculin to inhibit tyrosine phosphorylation and promote vinculin-mediated FA formation.

### CD147 affects focal adhesion dynamics

**Figure 5:**
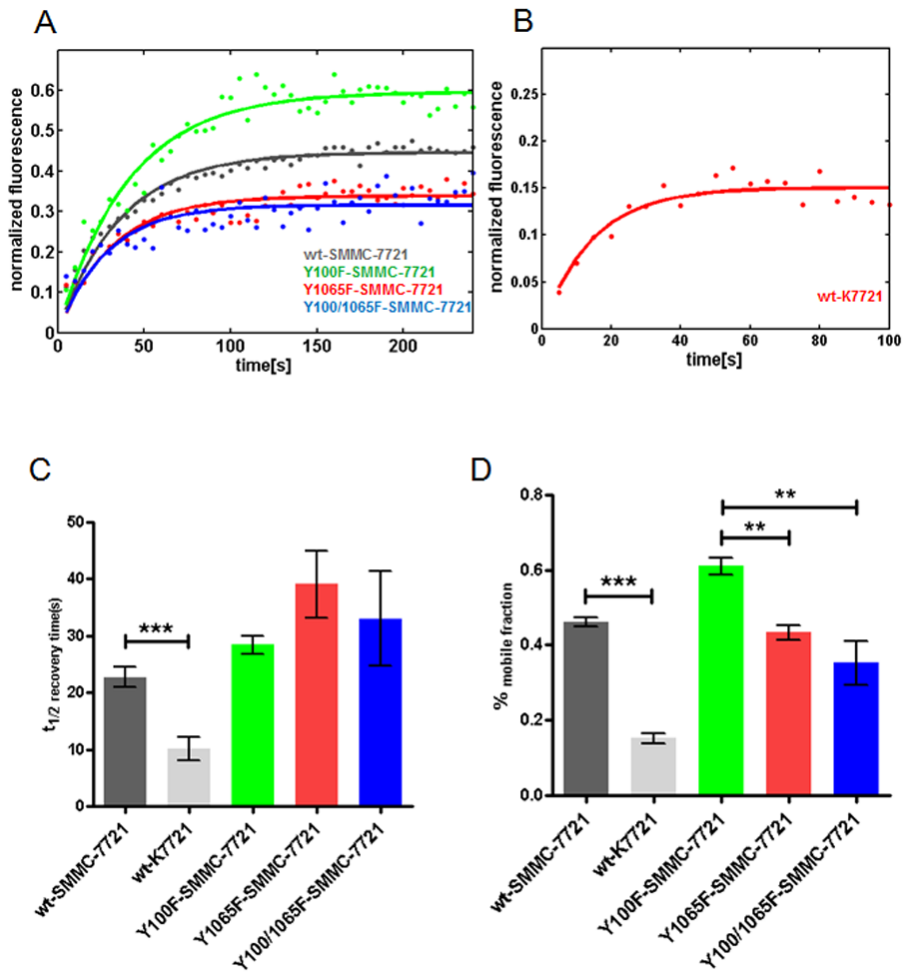
CD147 effects on vinculin-mediated FA dynamics. EYFP-tagged wt-vinculin and the vinculin mutants Y100F, Y1065F and Y100/1065F. (A–B) FRAP curves of EYFP-vinculin and mutants in SMMC-7721 and K7721 cells. Cells were subjected to FRAP experiments 16–24 h after plating at 37°C using a 60× oil objective (see Experimental Procedures). A single exponential model was fitted to these normalized fluorescence curves. Solid lines denote the best fit of a non-linear regression analysis. (C–D) Mean recovery halftimes and mobile fractions. The results represent the mean ± s.e.m. of 30–50 measurements. The mean half recovery times and mobile fraction of EYFP-tagged wt vinculin in SMMC-7721 cells are significantly different from those for K7721 cells. Error bars represent standard errors of the means. **P<0.001, ***P<0.0001 (Student’s *t*-test).

Assembly/disassembly dynamics are a primary characteristic of FAs. We used FRAP to evaluate the role of CD147 in vinculin-mediated FA dynamics. To examine FA recovery halftimes (*t*_1/2_) and mobile fractions (A), we constructed EYFP-tagged wild-type vinculin and mutants for different tyrosine phosphorylation sites (Y100F, Y1065F, and Y100F/Y1065F). As shown in Figs. 5A, B, and C, EYFP-tagged vinculin exhibited a longer recovery *t*_1/2_ (22.7±3.3 s) in SMMC-7721 cells than in K7721 cells (10.1±1.8 s), and the mobile fraction was relatively reduced when CD147 was deleted in K7721 cells (Fig. 5D). These results demonstrate that CD147 promotes vinculin-mediated FA protein interactions and recruitment.

The EYFP-conjugated Y100F and Y100/1065F mutants exhibited a smaller increase in FRAP *t*_1/2_ (28.3±1.6 s and 27.7±0.22 s, respectively) than EYFP-tagged wt-vinculin in SMMC-7721 cells. In contrast, the EYFP-tagged Y1065F mutant exhibited a longer recovery time (FRAP *t*_1/2_) than the other mutants. These results suggest that tyrosine phosphorylation of vinculin at Y1065 plays an important role in vinculin-mediated FA dynamics. The mobile fraction of the Y100F mutant increased significantly compared to Y1065F and Y100/1065F. This indicates that tyrosine 100 in the vinculin protein is important for cytoplasm protein recruitment.

### CD147 enhances cell spreading and HCC cell adhesion

**Figure 6:**
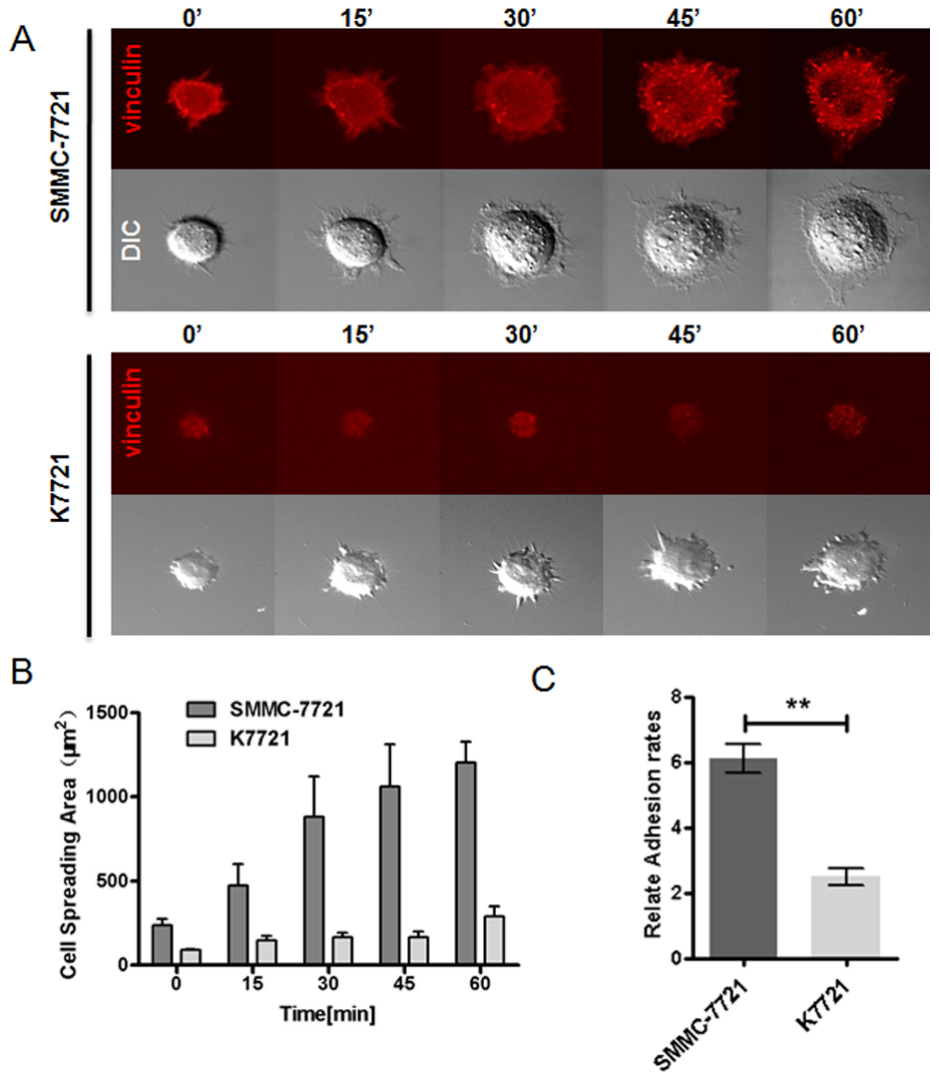
CD147 effects on spreading and adhesion of HCC cells. (A) Time-lapse imaging was used to analyzing the spreading of SMMC-7721 and K7721 cells. SMMC-7721 and K7721 cells were transfected with DsRed2-N1-vinculin to visual FA behavior. Confocal images were captured at different time points during cell spreading. (B) The cell spreading areas of SMMC-7721 and K7721 cells were determined with Nikon software. Data shown represent mean ± s.e.m. and are representative of three independent experiments. Error bars are standard errors of the means. (C) Adhesion potential of SMMC-7721 and K7721 cells. Cells were suspended in serum-free medium and seeded onto Matrigel-coated wells. After incubation for 30 min at 37°C, the percentage of adherent cells was determined using a toluidine blue assay. Data shown represent mean ± s.e.m. and are representative of three independent experiments. **P<0.001 (Student’s *t*-test).

Cell spreading is a distinct process that consists of passive adhesion and cell deformation [26]. During this process, FAs induce the rearrangement of F-actin to enable attachment to a substrate. We sought to determine whether CD147 regulates cell spreading by modulating vinculin-mediated FA formation in HCC cells. To visualize FA behavior during the cell spreading process, DsRed1-N1-vinculin was transfected into SMMC-7721 and K7721 cells. Time-lapse observations of the FA formation process were carried out during cell spreading. As shown in Fig. 6A, deletion of CD147 blocked the spreading of K7721 cells compared to SMMC-7721 cells. We then quantified FA areas in HCC cells. As shown in Fig. 6B, FA areas in K7721 cells were noticeably smaller than in SMMC-7721 cells (*P*<0.0001). This indicates that deletion of CD147 delays the cell-spreading process in HCC cells, and FA dynamics are severely impaired. Furthermore, deletion of CD147 reduced the adhesion rate of K7721 cells compared to SMMC-7721 cells (Fig. 6C).

## Discussion

In a previous study, we demonstrated that high expression of CD147 regulated focal adhesion signaling pathways and cytoskeleton reorganization to enhance invasion and metastasis in HCC cells. However, the precise mechanism was not determined.

One important biochemical characteristic of CD147 is its high level of glycosylation. N-glycans modulate many biological functions of CD147, including protein maturation and translocation to the cell membrane as well as facilitating oligomerization and thus promoting the production of MMPs [23]. Additionally, Li et al. found that the glycosylated form of CD147 is associated with differential lymphatic metastasis potential for murine hepatocarcinoma cells [27]. In the current study, evidence is presented that N-glycosylation of CD147 is required for redistribution of focal adhesions and actin cytoskeleton rearrangement, which occurs as HCCs undergo morphological changes during migration, implying that N-glycosylation of CD147 is also vital for cell migration as well as MMP production.

Although a previous study showed that vinculin-null cells exhibit smaller FAs and move more quickly than wild type cells [15], other studies have reported that vinculin facilitates invasion by up-regulating or enhancing the transmission of traction forces in a three-dimensional state [28]. In the present study, we found that high expression of CD147 correlated with larger FA areas. Based on our previous studies demonstrating that overexpression of CD147 promotes tumor invasion and migration, we suggest that tumor migration and invasion are complicated processes and that FA area is not the only factor that determines tumor migration.

Lamellipodia are sheet-like membrane protrusions at the leading edges of migrating cells [29]. The FA complex is organized by the Arp2/3-mediated actin filament branching network found at the leading edges of lamellipodia [30], [31]. Previous studies have shown that transient interaction of Arp2/3 with vinculin is regulated by PI3K and Rac1, which recruit the Arp2/3 complex to new sites of integrin clustering [30], [31]. Our previous work indicated that CD147 promotes PI3K and Rac1 activation to induce lamellipodia formation [12]. The present study shows that deletion of CD147 reduces the accumulation of Arp2/3 at the cell edge, and inhibits lamellipodia formation in HCC cells. This suggests that CD147 is essential for the formation of lamellipodia protrusions in motile HCC cells.

FAK is a non-receptor tyrosine kinase that plays a major role in integrin signaling by forming a complex, and regulating the tyrosine phosphorylation of FA proteins [32]. Furthermore, FAK was shown to be involved in FA turnover dynamics [33]. Our previous studies indicated that CD147 interacts with integrins and up-regulates FAK expression and phosphorylation levels [10]. The present study demonstrates that CD147 reduces vinculin-mediated tyrosine phosphorylation levels to promote FA stability and maturation. This is consistent with other studies that showed most vinculin proteins are dephosphorylated in FAs [34] and that FAK modulates vinculin recruitment to the FA complex [35]. Thus, we suggest that CD147 is involved in the dynamic assembly of FAK-regulated and vinculin-mediated FAs in HCC cells.

Vinculin is a stable marker of FAs and exists in equilibrium between its activated and inactivated states [36]. Folded forms of vinculin in an inactivated state exist in the cytoplasm, whereas the activated forms only reside in focal adhesions [37]. PIP2 disrupts the head–tail interactions between vinculin molecules and activates this protein [25], [38], [39]. In the current study, we showed that CD147 promotes PIP2 accumulation in HCC cells. This potentially explains why vinculin-mediated FA areas are larger in SMMC-7721 cells than in K7721 cells, in which CD147 is deleted.

Dynamic assembly and disassembly are among the most significant features of FAs. Using the FRAP technique and mathematical analysis, we demonstrated that tyrosine phosphorylation of vinculin plays an important role in vinculin-mediated FA dynamics. Interaction between CD147 and vinculin led to decreased tyrosine phosphorylation of vinculin and more stable and larger vinculin-mediated FAs. Vinculin pTyr mutants (Y100F, Y1065F and Y100/1065F) exhibited severely impaired interactions between CD147 and vinculin. This suggests that the tyrosine phosphorylation sites of vinculin are critical to permit this interaction and thus regulate vinculin-mediated FA dynamics. The cell spreading assay also revealed normal dynamic cell spreading when CD147 was expressed. This indicates that CD147 acts to strengthen the connection between FAs and F-actin and to induce reorganization of the cytoskeleton to facilitate cell migration. Although, our results demonstrated that CD147 plays a role in FA formation and dynamic reorganization of the cytoskeleton, these results were performed on 2D cultured HCC cells. A 3D cell culture model might have been a closer simulation to an actual ECM environment. In the future, we plan to study CD147 function, and its relationship to FA and the cytoskeleton in various matrices and in 3D cultured cells. The FA consists of dozens of proteins. FAK, talin, and paxillin are important for FA dynamics and morphology. The synergistic effect of these proteins should be investigated further. Proteomics technology should be used for identification these up-regulated proteins. We suggest that these proteins are associated with CD147 overexpression during tumor invasion and metastasis. However, the mechanism by which CD147 regulates vinculin-FA should be tested in tumor cells. Future experiments are planned to further study the mechanism of CD147 in regulation of FA properties and cytoskeleton rearrangement.

In summary, our study demonstrated that CD147 is critical for lamellipodia formation via the activation of the Arp2/3 complex. We also showed that the interaction between CD147 and vinculin reduces tyrosine phosphorylation and dynamically regulates the assembly of focal adhesions and cytoskeleton organization to facilitate HCC cell migration. Therefore, CD147 is an important protein for cancer cell migration, and an attractive target for CD147 antagonists as antitumor treatment.

## Supporting Information

Figure S1 FRET imaging of the interactions between vinculin mutants and CD147. FRET analysis was performed as described in the protocol for Fig. 4B.
(TIF)
