# Supplementary figures and images for: HAb18G/CD147 Regulates Vinculin-Mediated Focal Adhesion and Cytoskeleton Organization in Cultured Human Hepatocellular Carcinoma Cells
Source: PLoS One. 2014 Jul 17;9(7):e102496. doi: 10.1371/journal.pone.0102496 (PMC4102505; doi:10.1371/journal.pone.0102496)

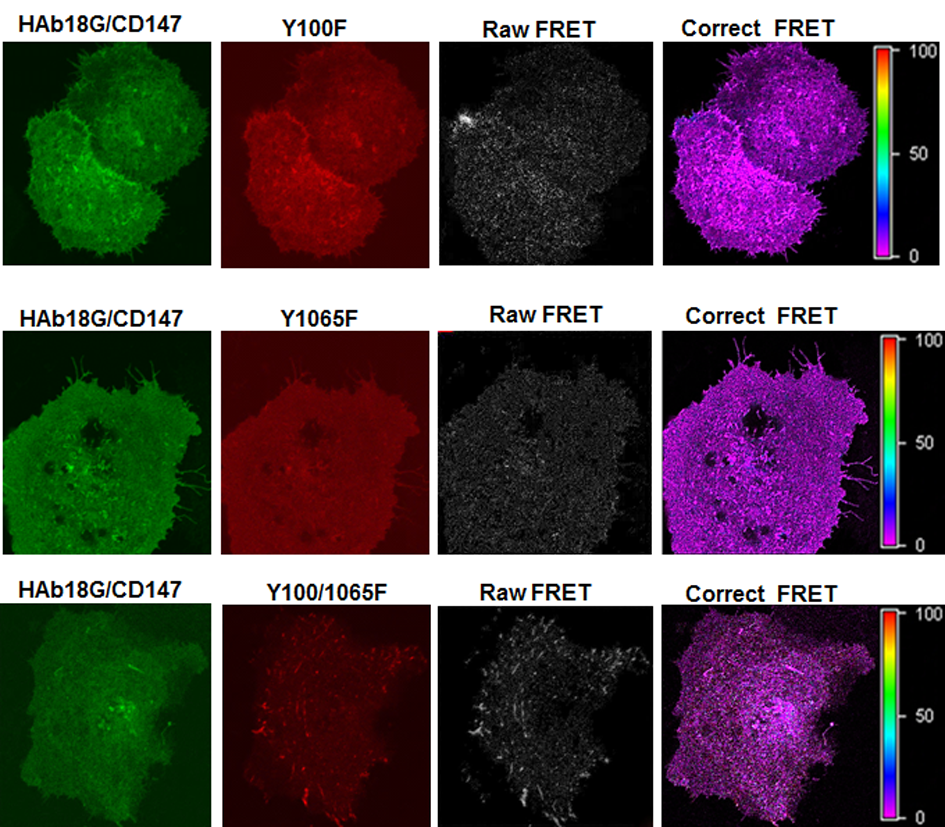

Supplement: Figure S1 — FRET imaging of the interactions between vinculin mutants and CD147. FRET analysis was performed as described in the protocol for Fig. 4B. (TIF) [file pone.0102496.s001.tif]
